# Editorial: Pharmaceutical strategies to prevent, treat, and recover: advances and challenges in ischemic stroke and hemorrhagic stroke

**DOI:** 10.3389/fnins.2024.1383941

**Published:** 2024-02-21

**Authors:** Linqiao Tang, Tiejun Zhang, Gang-Min Hur, Yuwen Li

**Affiliations:** ^1^Department of Pharmacy, West China Hospital, Sichuan University, Chengdu, China; ^2^Research Core Facility, West China Hospital, Sichuan University, Chengdu, China; ^3^Department of Neurosurgery, West China Hospital, Sichuan University, Chengdu, China; ^4^Department of Pharmacology, Research Institute for Medical Science, College of Medicine, Chungnam National University, Daejeon, Republic of Korea

**Keywords:** stroke, cerebrovascular disease, clinical management, neuropharmacology, neuroprotection

Stroke stands as one of the primary causes of death and disability globally, particularly with an increasing incidence in developing countries (Campbell et al., 2019). At the present topic, Zhou et al. documented a fatality resulting from cerebral hemorrhage after parathyroid surgery, underscoring the existence of obscure yet highly lethal etiological factors. Complications arising from strokes are prevalent and pose significant threats to life. Zheng M. et al. emphasized the need to consider lumbar cistern blockage as a potential complication following cerebral hemorrhage. However, pharmaceutical therapies for both ischemic and hemorrhagic stroke are still lacking, and the available therapeutic strategies are generally time-sensitive (Jovin et al., 2022). Thus, there is an urgent need to explore the complex regulation network after stroke, which is essential for developing new generations of effective treatment strategies. Besides, translational research and clinical trials are encouraged to improve the prognosis of stroke in the future. This Research Topic includes 17 articles concerning pathological mechanisms, new therapeutic entities, and other attractive aspects of stroke.

Concurrently, stroke research remains at the forefront of technological advancements. Qiu et al. presented a novel method for identifying differentially expressed genes in spatial transcriptomics data, exemplifying the integration of cutting-edge technologies in stroke investigations. The intricate relationship between brain function and structure is elucidated through spatial-omics techniques like spatial transcriptomics, providing researchers with a more precise characterization of biological processes with spatial data. Recognizing the pivotal role of stem cells in brain function recovery (van Velthoven et al., 2013; Bacigaluppi et al., 2016; Tornero et al., 2017), Zhang Q. et al. provided a comprehensive summary of the progress in stem cell applications within the realm of stroke research, offering valuable insights into their potential therapeutic utility.

The pathological changes following the onset of a stroke are intricate, involving the activation/inactivation of multiple cells and pathways, extending even to the interplay between the brain and gut microbiota (Jeong et al., 2023). These complexities render stroke treatment a formidable task, and various articles on this topic have synthesized novel insights into targets and therapeutic strategies for stroke. Wang J. et al. reviewed approaches such as dietary intervention, fecal microbiota transplantation, probiotics, antibiotics, traditional Chinese medication, and gut-derived stem cell transplantation were explored for their influence on gut microbiota and subsequent regulation of brain recovery post-stroke. Yuan et al. delved into intermittent hypoxic conditions as a potential strategy for ischemic stroke prevention and treatment. Their examination encompassed current evidence, future research directions, and the proposal of intermittent hypoxia as a non-invasive, non-pharmacological, systemic, and multi-targeted intervention to mitigate brain damage.

Contemporary drug research and development are predicated on the identification of specific targets. Two studies included in this topic have uncovered novel druggable targets within this context. Zuo et al. employed CXC3CR1+/GFP mice to elucidated the increased expression of myeloperoxidase (MPO) on microglia after ICH. This MPO-targeted therapeutic interventions consequently facilitated the restoration of motor function. Meanwhile, Zhang C. et al. offer a comprehensive overview of the versatile roles played by 15 specific lncRNAs in the pathological alterations associated with ICH, suggesting valuable insights into potential drug research and development targets.

Traditional Chinese medicine (TCM), often composed of multiple herbs, represents a repository of numerous chemical entities with potential efficacy in stroke treatment. Zheng L. et al. conducted a comprehensive review of studies on treating stroke with *Sanhua decoction*, consisting of *Polygonaceae, Magnoliaceae, Rutaceae*, and *Apiaceae*, and demonstrated its multiple effects on brain repair. Similarly, Li L. et al. analyzed the potential active pharmaceutical ingredients of another TCM, *Zuogui Pill*, which facilitated neurite outgrowth via mTOR, p53 and Wnt signaling pathways following ischemic stroke. A pressing need persists for high-level evidence regarding herbal medicines, herbal extracts, gut microbiota, or any novel strategy for treating stroke (Zheng L. et al.). This need is pivotal for translating preclinical findings into clinically applicable interventions. Consequently, numerous scholars actively investigate the safety and efficacy of herbal medicines or extracts in stroke treatment through well-designed clinical trials. For instance, a multi-center, open-label, pilot randomized clinical trial conducted by Cui et al. demonstrated the effectiveness of *Ginkgo biloba* extract in protecting patients from cognitive decline 24 weeks after acute ischemic stroke, as assessed through various evaluation methods.

Additionally, specific chemical entities and therapeutic modalities have gained noteworthy attention within stroke research delineated the attributes of TJ-M2010-5, a BBB-permeable drug candidate exhibiting efficacy as a MyD88/NF-κB and ERK pathway inhibitor (Li Z. et al.). Wang X. et al. investigated the systemic administration of dobutamine, revealing its capacity to expedite erythrocyte clearance from the brain to cervical lymph nodes following SAH. Intriguingly, Pichardo-Rojas et al. conducted a comprehensive review highlighting memantine's ability to mitigate NMDA-mediated excitotoxicity, preserve intracellular ATP stores, and up-regulate neuron-specific growth factor expression, with clinical evidence proving its neuroprotective effects in ICH, ischemic stroke, and ischemic stroke-related aphasia. Besides, anesthesias utilized during endovascular thrombectomy (EVT), including ketamine, propofol, sevoflurane, and isoflurane, have been identified as beneficial for post-stroke brain protection (Zhang T. et al.).

Moreover, this topic includes two meta-analyses evaluating the efficacy of inclisiran and mechanical thrombectomy in stroke. Luo et al. analyzed three randomized clinical trials (ORION-9, ORION-10, and ORION-11), concluding that inclisiran did not exhibit significant effects in preventing stroke in atherosclerotic cardiovascular disease patients or those at high risk. Meanwhile, Yang et al. analyzed seven studies (including 1,083 patients) and observed that mechanical thrombectomy with intra-arterial alteplase might enhance functional outcomes without significantly impacting recanalization. These meta-analyses contribute valuable insights to inform clinical decision-making processes.

The assortment of manuscripts within this Research Topic underscores the diverse facets of stroke prevention, treatment, and recovery. These articles contribute novel perspectives to preclinical research and expand the horizons of clinical practice. Notably, the impact of TCM, intestinal microbiota, intermittent hypoxia, and anesthetic medications on stroke prognosis is particularly enlightening. This underscores that, while stroke remains a condition associated with high mortality and disability rates, preventive and therapeutic tools are at our disposal. This realization catalyzes advancing translational research to translate cutting-edge findings in stroke research into clinical applications expeditiously.

We eagerly anticipate further revelations, discussions, and practical applications in emerging areas, such as new drug delivery systems. These developments promise to unveil additional possibilities for stroke prevention, treatment, and recovery, paving the way for continued advancements in stroke-related research and clinical interventions.

## Author contributions

LT: Writing—original draft, Writing—review & editing. TZ: Writing—review & editing. G-MH: Writing—review & editing. YL: Writing—review & editing.
